# The Chromosomal High-Affinity Binding Sites for the *Drosophila* Dosage Compensation Complex

**DOI:** 10.1371/journal.pgen.1000302

**Published:** 2008-12-12

**Authors:** Tobias Straub, Charlotte Grimaud, Gregor D. Gilfillan, Angelika Mitterweger, Peter B. Becker

**Affiliations:** Molecular Biology Unit and Centre for Integrated Protein Science (CiPSM), Adolf-Butenandt Institute, Ludwig-Maximilians University, Munich, Germany; Massachusetts General Hospital, Howard Hughes Medical Institute, United States of America

## Abstract

Dosage compensation in male *Drosophila* relies on the X chromosome–specific recruitment of a chromatin-modifying machinery, the dosage compensation complex (DCC). The principles that assure selective targeting of the DCC are unknown. According to a prevalent model, X chromosome targeting is initiated by recruitment of the DCC core components, MSL1 and MSL2, to a limited number of so-called “high-affinity sites” (HAS). Only very few such sites are known at the DNA sequence level, which has precluded the definition of DCC targeting principles. Combining RNA interference against DCC subunits, limited crosslinking, and chromatin immunoprecipitation coupled to probing high-resolution DNA microarrays, we identified a set of 131 HAS for MSL1 and MSL2 and confirmed their properties by various means. The HAS sites are distributed all over the X chromosome and are functionally important, since the extent of dosage compensation of a given gene and its proximity to a HAS are positively correlated. The sites are mainly located on non-coding parts of genes and predominantly map to regions that are devoid of nucleosomes. In contrast, the bulk of DCC binding is in coding regions and is marked by histone H3K36 methylation. Within the HAS, repetitive DNA sequences mainly based on GA and CA dinucleotides are enriched. Interestingly, DCC subcomplexes bind a small number of autosomal locations with similar features.

## Author Summary

In sexually dimorphic species, unequal distribution of sex chromosomes requires adjustment of gene expression levels between the sexes. Male flies enhance transcription from the single X chromosome to meet the levels in females (XX). The specific recognition of sex chromosomes is a crucial step in this dosage compensation process. Intuitively, one might assume that sex chromosomes harbor distinct DNA sequence motifs for recruitment of the modulating machinery; however, no clearly defined motifs capable of fulfilling this role have yet been found. One explanation for this shortcoming could be our failure to date to identify a sufficiently large set of sites that serve as specific docking stations. In the following study, we have systematically mapped the strongest recruitment sites of the *Drosophila* dosage compensation complex (DCC) and identified shared sequence elements. The closer a gene resides to one of these sites the more robust is regulation by the DCC, which documents the function of our inventory of high-affinity binding sites.

## Introduction

Genes residing on the single X chromosome in male *Drosophila* flies are transcribed at elevated rates to match the expression levels of the two X chromosomes in female cells. Transcriptional tuning in male cells depends on the activity of a ribonucleoprotein complex, the dosage compensation complex (DCC, also referred to as MSL [male-specific lethal] complex, reviewed in [1],[2]). Formation of DCC is male-specific due to the expression of the key subunit MSL2, which in turn drives the expression of the non-coding RNA components of the DCC, the roX (RNA on the X) RNAs [3],[4]. The complex associates almost exclusively with the X chromosome, which explains the selective activation of X chromosomal genes. This is at least in part due to the acetylation of lysine 16 of histone H4 (H4K16) by the histone acetyltransferase (HAT) MOF, an integral subunit of the DCC [5]. This modification may directly lead to unfolding of the chromatin fiber [6] or indirectly counteract factors that promote the formation of repressive chromatin [7],[8] rendering chromatin more permissive to the progress of transcription.

The phenomenon of dosage compensation allows the study of general principles of transcriptional fine-tuning and chromosome-wide regulation. A key question is how the DCC is recruited specifically to the X chromosome. High-resolution mapping demonstrated that the complex targets transcriptionally active regions on the X chromosome with a preference for coding sequences [9],[10]. The DCC distribution pattern cannot be easily explained by a single targeting principle, but presumably results from the successive application of two or more distinct principles. Early genetic experiments led to a concept that assumes the existence of a relatively small number of X chromosome-specific primary recruitment or chromosomal ‘entry’ sites (CES) for the DCC, from which the complex would ‘spread’ to the bulk of chromosomal binding sites that differ qualitatively from the entry sites [11],[12]. Entry sites could, for example, be defined by a particular DNA sequence element, whereas features of active chromatin combined with proximity to entry sites would be a hallmark of secondary sites. Subsequent studies disputed whether DCC binding sites should be sorted into categories defined by different recruitment principles, or whether all targeting could be explained by a single principle (e.g. DNA sequence) that was applied to define sites of higher or lower affinity [13],[14],[15],[16]. Independent of whether primary recruitment sites differ from the bulk of DCC binding sites in quality or by a quantitative feature, they attract the DCC under stringent conditions. For example, DCC is recruited to high-affinity sites (HAS) even if they are removed from the X chromosomal context and inserted on an autosome, or at low levels of DCC (genetically achieved through expression of low amounts of MSL2) [16],[17], or if the integral DCC subunits MSL3, MLE, MOF or the roX RNAs are absent [18],[19],[20]. Under the latter circumstances binding sites are demarcated by binding of a sub-complex consisting of only MSL1 and MSL2 [19]. Evidently, distribution of DCC to sites of supposedly lower affinity depends on MOF, MSL3, MLE and the roX RNAs.

More recently, Kuroda and colleagues obtained additional support for the concept that primary and secondary DCC binding sites are defined by different principles by showing that the binding to active chromatin in the vicinity of primary targeting sites is not X-specific [21]. Insertion of a *roX* gene in an autosome leads to extended ‘spreading’ of the DCC over the neighboring chromatin (both *roX* genes contain a HAS [11],[22]). Under these circumstances, the DCC associated with transcribed sequences on autosomes like it normally does on the X chromosome. Recruitment of DCC was suggested to involve binding to methylated histone H3 at lysine 36 (H3K36me3), a modification that is placed by histone methyltransferases associated with elongating RNA polymerase II (pol II) and hence marks sites of active transcription [21].

X chromosome-specific targeting may, therefore, be encoded by primary targeting sites. So far, just a few DNA elements that robustly fulfill the criteria for a primary targeting site have been characterized at the DNA sequence level. These include sites within the roX genes [22],[23], the Smr and Tao-1 genes [13] as well as a site that maps to cytological position 18D [15]. Due to this limited number, a defining feature with predictive value could not be extracted, although the presence of multiple distinct DNA sequence elements has been correlated with HAS [22],[24]. Strikingly, low complexity sequence elements such as GA- and CA-based dinucleotide repeats as well as runs of adenines have repeatedly been noted in these analyses [10],[13],[24]. Dissection of HAS DNA has yielded sub-fragments that retain limited binding activity. We therefore suggested that primary targeting is based on the local clustering of distinct sequence motifs [13],[24].

Progress on HAS definition requires moving the analysis from the anecdotal to the systematic level. We therefore mapped all DCC binding sites with highest affinities on a chromosome-wide scale by combining chromatin immunoprecipitation (ChIP) with probing of high-resolution DNA tiling arrays (ChIP-on-chip) under conditions where sites of higher affinity are preferentially visualized. This strategy not only allowed the generation of a sufficiently large training set for sequence analysis, but at the same time provided a means to directly compare the high affinity binding pattern with several chromatin features that have already been mapped along the *Drosophila* male X chromosome.

## Results

We followed two complementary strategies for filtering the DCC binding sites with highest affinity from the chromosome-wide binding profile. First, we attempted to reproduce in male tissue culture cells the conditions that lead to selective visualization of HAS on polytene chromosomes in mutant larvae, where MSL1 and MSL2 interact selectively with HAS in the absence of MSL3, MLE or MOF [13],[18],[19]. Towards this goal we reduced the levels of these factors in the male *Drosophila* cell line SL2 by RNA interference (RNAi) and monitored the residual interaction of MSL1 or MSL2 (genetic studies have established the mutual interdependence of these two subunits for their interactions with the bulk of X chromosomal sites [19]). The second strategy followed the idea that HAS should, on average, show a higher occupancy by DCC and hence should be selectively obtained by ChIP if the extent of formaldehyde crosslinking was reduced. Lower levels of crosslinking should also reveal sites of more intimate contact of MSL proteins with DNA. Reassuringly, both strategies led to a similar alteration of the MSL binding pattern with enhanced peaks along all previously known HAS. The combined data should therefore help to define an inventory of sites with similar properties.

### Coding Sequences Have the Least Affinity for the DCC

**Figure 1:**
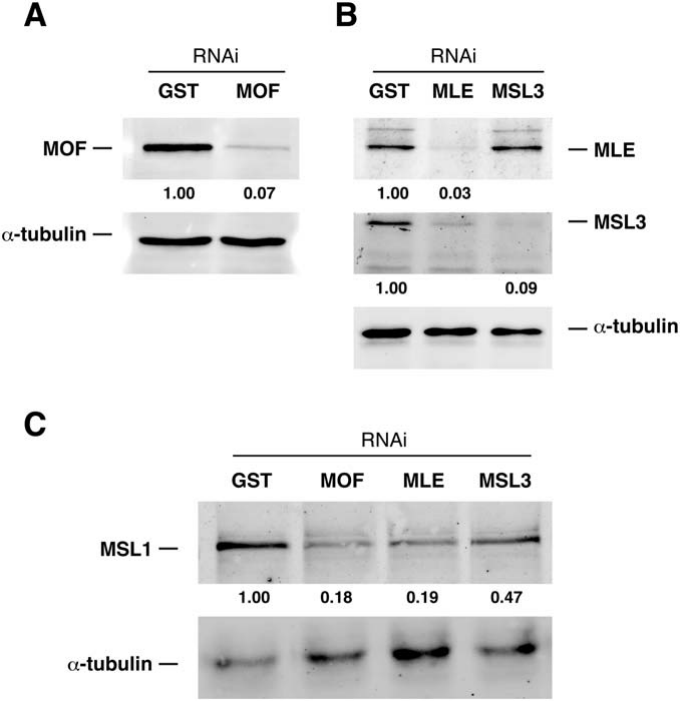
Confirmation of RNAi target depletion by western blotting. Effect of (A) MOF RNAi on MOF and (B) MLE and MSL3 RNAi on corresponding protein levels. Relative amounts of target protein after interference normalized to α-tubulin are indicated. C) Effect of RNAi on MSL1 protein levels. Relative amounts of MSL1 are indicated.

We lowered the levels of MSL3, MLE and MOF in SL2 cells by RNAi and mapped the residual binding pattern of the DCC core components MSL1 and MSL2 by ChIP-on-chip. All knock-down experiments were controlled for non-specific effects by a parallel RNAi treatment with an irrelevant dsRNA (which corresponds to glutathione-S-transferase (GST) sequences: ‘GST RNAi’). After 7 days of treatment with double-stranded (ds) RNA we achieved approx. 90% depletion of the target proteins as compared to the RNAi GST control (Figures 1A & B). Removal of MLE also led to reduction of MSL3 levels. Furthermore, RNAi-mediated depletion of MLE, MSL3 and MOF resulted in a substantial reduction of MSL1 to as little as 20%, indicating a global destabilization of the complex (Figure 1C). A similar drop in protein levels was observed for MSL2 (not shown). These circumstances should further facilitate selecting binding sites of only the highest affinity.

In flies a genetic knockout of MLE or MSL3 leads to the most pronounced reduction of MSL1-MSL2 binding [13]. We therefore first investigated the residual MSL1 profile after RNAi against MLE or MSL3 (Figure S1). The chromosomal interaction profile showed surprisingly mild effects: only 6 and 9% of all significant MSL1 binding events were lost upon MSL3 or MLE depletion, respectively. On all MSL1 target probes we observed a moderate reduction of MSL1 signals (Figure S1C). Conceivably, the remaining DCC subunits after incomplete knockdown may suffice to sustain MSL1 binding. However, in light of results from the analysis of mutant fly strains we consider more likely that the ChIP-on-chip methodology underestimates homogeneous inter-array differences and therefore might obscure a global reduction of MSL1 binding under knockdown conditions. This would be due to disproportional procedures such as array hybridization and scanning as well as signal normalization across arrays. Visual inspection of the binding pattern, however, allowed for the identification of loci where MSL1 association was substantially reduced (such as the small gene cluster in the right half of Figure S1A). This indicates, that MSL3 and MLE RNAi cause a local redistribution of MSL1, which should contribute to the identification of high affinity regions that are supposed to be more resistant to these perturbations. In the case of MSL3, examination of the loss of MSL1 binding within distinct functional regions revealed a significantly stronger reduction in coding sequences as compared to other binding regions (p-value<2.2e-16; two-sided t-test; Figure S1C, right green box).

**Figure 2:**
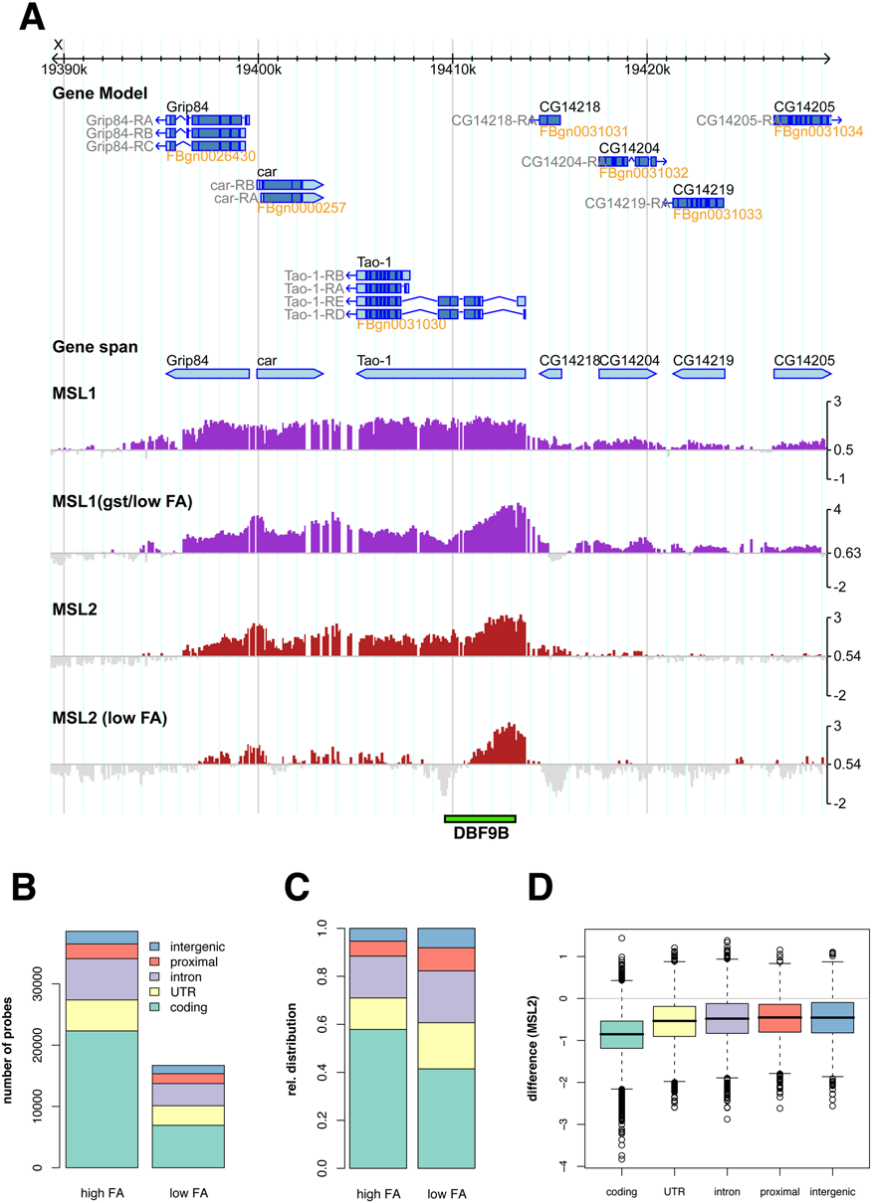
Differential crosslinking alters the binding patterns and improves mapping of high-affinity sites. A) Genome browser snapshot with gene spans and gene models. MSL1 and MSL2 profiles after low or high formaldehyde (FA) crosslinking are depicted as the log2 of the mean enrichment ratio (IP/Input) of at least 2 replicate experiments. The *Tao-1* gene contains a confirmed HAS (DBF 9B), which is indicated by the green box below the profiles. B) Absolute changes in numbers of probes significantly bound by MSL2 after differential crosslinking. C) Corresponding relative changes according to functional context. D) Changes in MSL2 signals on MSL2 target probes grouped according to genomic context when crosslinking under low FA conditions compared to high FA crosslinking.

We next explored the usefulness of our second strategy and established the MSL2 binding profile at lower levels of formaldehyde crosslinking. We fixed cells with only 0.1% instead of 1% of formaldehyde (see Materials and Methods for details). Crosslinking with low concentrations of formaldehyde, we lost about 50% of significant MSL2 binding (Figure 2B), which preferentially affected coding sequences (p-value<2.2e-16; two-sided t-test; Figures 2C and D). We were encouraged by the fact that all genetically identified HAS were retained among the residual MSL2 peaks. For example, a previously identified HAS within the first intron of the Tao-1 gene coincides with a pronounced MSL2 peak (Figure 2A; [13],[24]).

**Figure 3:**
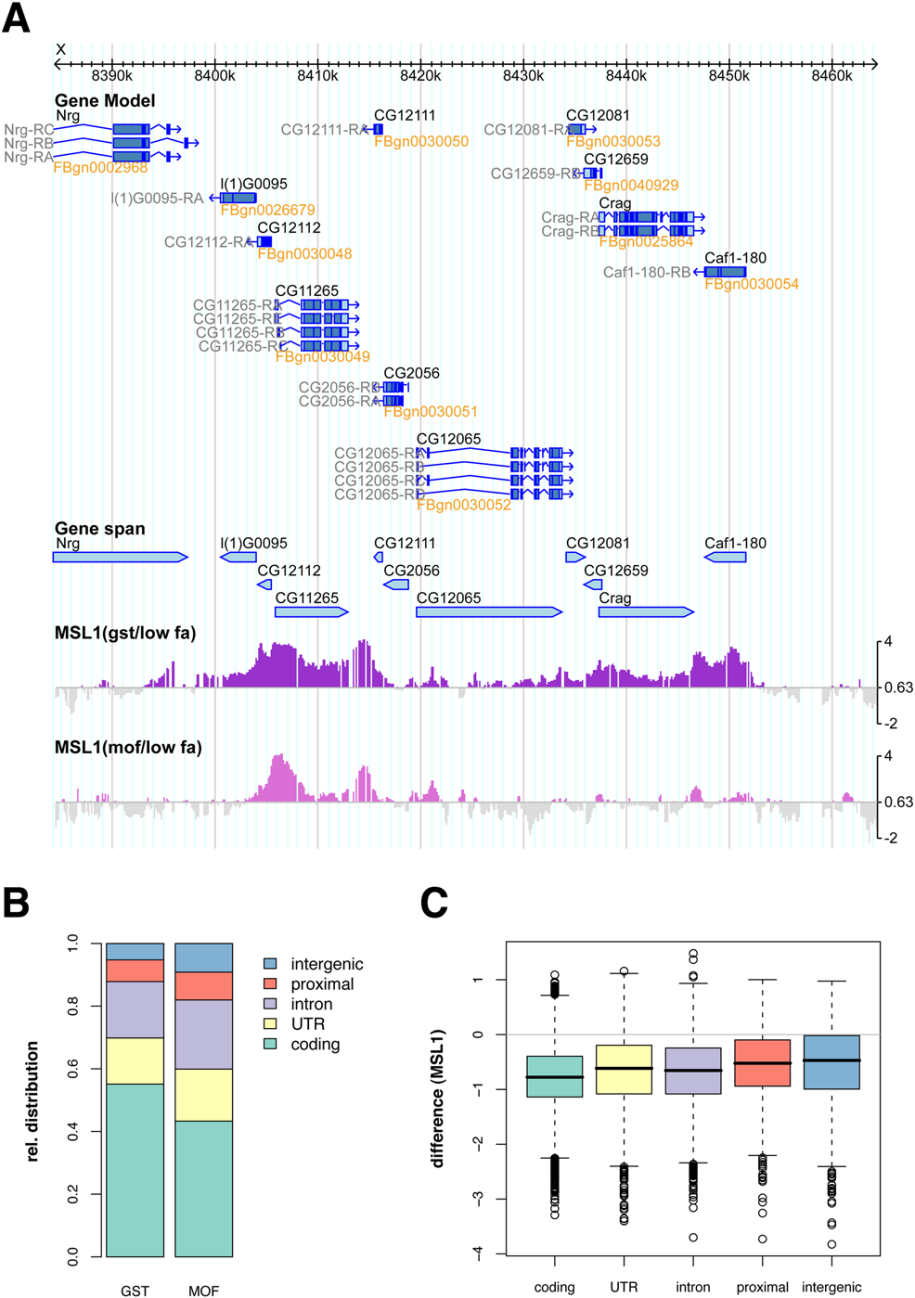
Changes in MSL1 binding after MOF RNAi. A) Genome browser snapshot of a representative region with MSL1 profiles after control (top) and MOF (bottom) RNAi. B) Changes of the relative distribution of significant MSL1 binding. C) Changes in MSL1 signal after MOF RNAi as compared to the signals after GST RNAi. Probes are grouped according to their functional annotation.

We then applied the same crosslinking conditions to the analysis of MSL1 binding after MOF RNAi (Figure 3). Comparing the pattern to the control pattern obtained after RNAi with GST sequences, the global reduction of MSL1 interaction across all genomic regions was much more pronounced than in the case of RNAi against MSL3 or MLE (compare Figure S1C to Figure 3C). The GST RNAi control sample that was also fixed with low formaldehyde exhibited similar alterations of the MSL1 binding pattern as observed for MSL2 (Figure 2A). Depletion of MOF resulted in loss of 23% of all significant binding events, again with a strong preference for coding sequences (p-value<2.2e-16; two-sided t-test; Figures 3B and C).

In summary, we found that two unrelated strategies aimed at selecting DCC binding sites of higher affinities led to an overall reduction of chromosomal association of the core DCC components MSL1 and MSL2 with a preferential loss of binding from coding regions. We tentatively conclude that coding regions are less likely to contain HAS.

### High-Affinity Chromosomal DCC Interaction Sites Are Mainly Located on Non-Coding Parts of Genes

**Figure 4:**
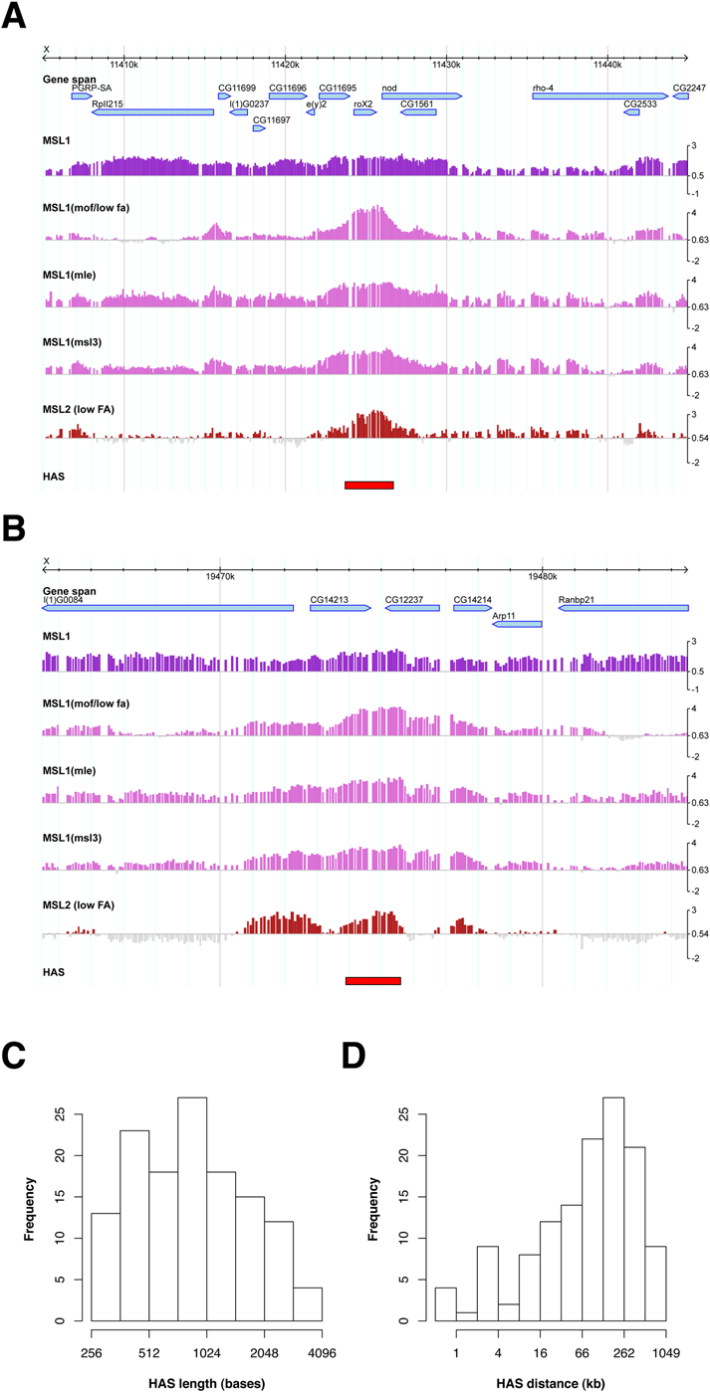
The new set of 131 X-chromosomal sites contain all established high-affinity sites of the Drosophila DCC. A) rox2 and B) the site at 18D displayed with MSL1 wild type and residual profiles after RNAi and/or low formaldehyde crosslinking. Histograms of the length distribution (C) and distance distribution (D) between high-affinity sites (histograms were calculated on log-transformed values).

We then attempted to identify particular genomic regions that are similarly enriched under both experimental regimes: upon RNAi against the spreading factors and at low levels of crosslinking. We transformed the enrichment ratios of all residual profiles (i.e. MSL1 binding after RNAi against MOF, MLE, or MSL3; MSL2 binding after low crosslinking) to z-scores and calculated an unweighted cumulative z-score. Region thresholding on the smoothed z-score profile allowing for a maximum of 1% autosomal site detection identified 130 HAS spread all along the X chromosome (Table S1). In addition, this approach picked up one autosomal site. The median length of the sites is 800 bases and the distribution of their distances peaks between 130 and 260 kb (Figures 4C and D). Even though some of the RNAi experiments had a limited effect on the global MSL1 distribution, it turned out that all caused local redistributions of MSL1 signals with a significant retention on HAS (Figure S2).

**Figure 5:**
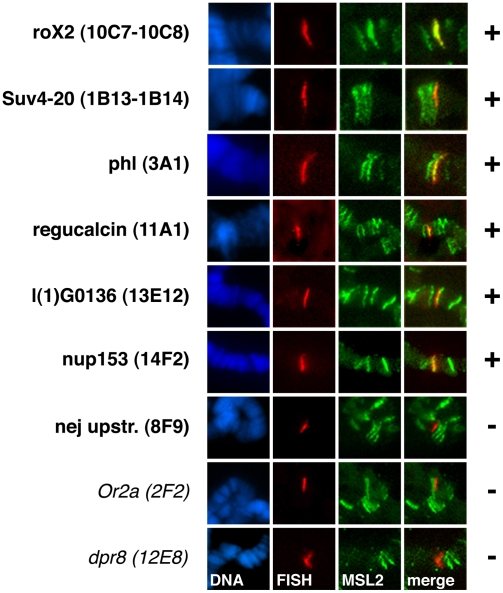
High-affinity sites in SL2 cells frequently map to MSL2 signals in Sxb1-2C females by Immuno-FISH on polytene chromosomes. Each row corresponds to one locus and is labeled with the name of the closest gene and the cytological position. (+) on the right indicates that FISH probe signal and MSL2 colocalized robustly; in the case of (−) no colocalization could be observed. No colocalization is expected with the control FISH probes *Or2a* and *dpr8*.

Our set contains all genetically defined robust HAS (roX1, roX2, 18D, Smr and Tao-1 [11],[13],[15]). For example, Figure 4 documents the correspondence of the previously mapped HAS within the roX2 gene (Figure 4A) and the HAS at 18D (Figure 4B). To test this correspondence more generally we picked six new HAS and mapped them with respect to the ‘entry sites’ visualized on polytene chromosomes by Immuno-FISH. We chose female larvae of the Sxb1-2C line, which express low levels of MSL2. The reduced amount of DCC in these flies binds to a small number of chromosomal loci that coincide with the ones observed in males mutant for *mle*, *msl3* or *mof* [13]. Larvae of this fly strain have previously been used to define ‘entry’ sites and to characterize HAS [13],[16]. Five out of six sites robustly colocalized with MSL2 binding sites (Figure 5). One of our strongest sites upstream of the *Nej* gene did, however, not show an overlap with the MSL2 pattern (see discussion). In contrast, control FISH probes located 60 kb (*Or2a*) or 400 kb (*dpr8*) away from the next HAS did not colocalize with MSL2.

**Figure 6:**
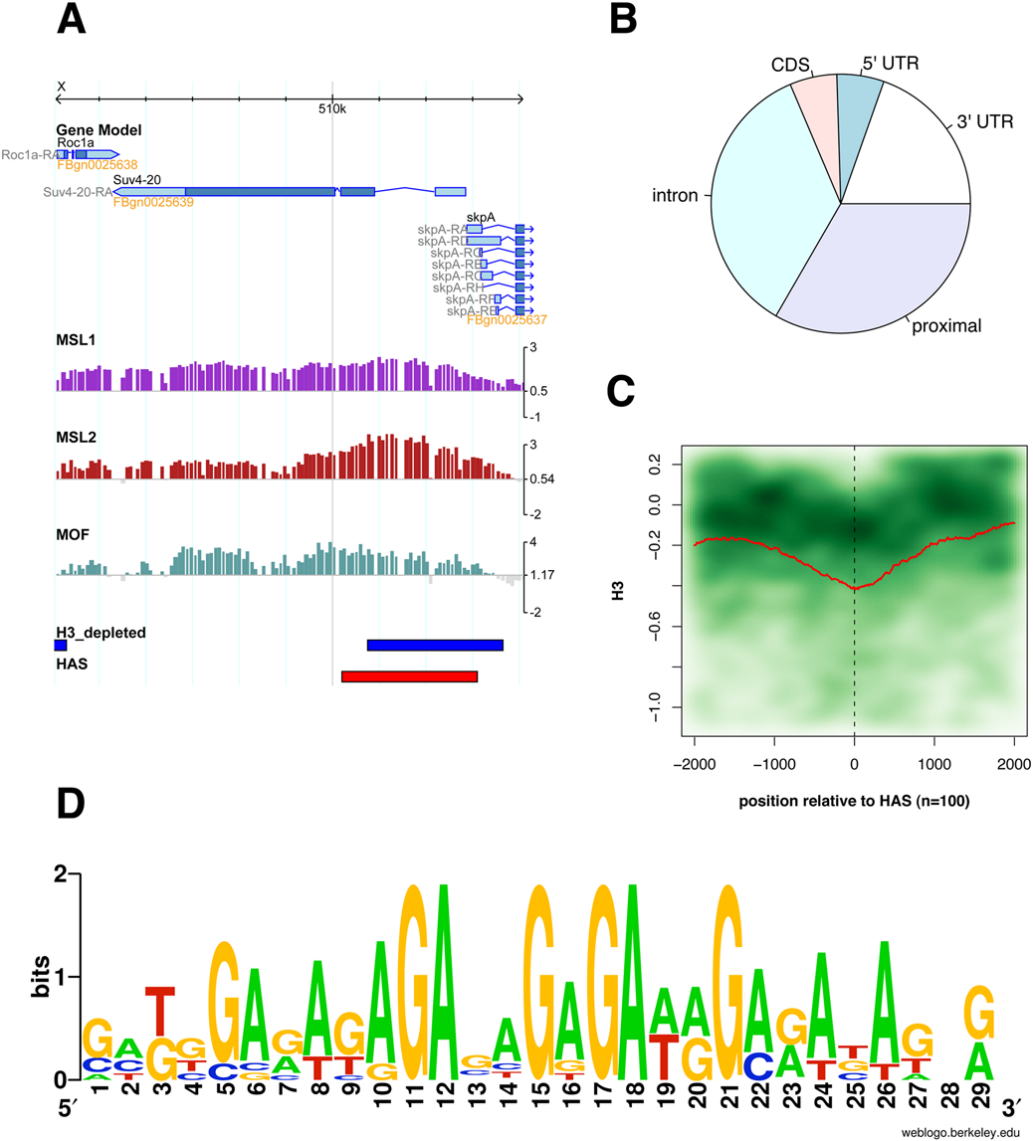
Common features of X-chromosomal high-affinity sites. A) A high affinity site (HAS, red box) within the *Suv4-20* gene. Displayed are wild type profiles for MSL1, MSL2 and MOF. Regions that are significantly depleted of histone H3 are indicated by blue boxes. B) Location of 51 high-affinity sites with respect to functional genomic context. “Proximal” indicates location within 3 kb up- or downstream of annotated genes. C) Cumulative H3 profile of 100 high-affinity sites. The red line indicates the mean signals along a region from −2 kb to +2 kb from the center of each high affinity site. The green cloud resembles the kernel density plot of all signals contributing to the mean. A darker color indicates higher density. D) Top motif found enriched in high-affinity sites and 1.5-fold over-represented on the X chromosome.

We next explored whether HAS location could be attributed to a particular functional context (intergenic, UTR, intron, coding sequences, etc). Many of the 131 sites are too large for precise functional assignment, since they contain coding as well as non-coding sequences. However, we found 51 regions that are unambiguously located within a defined functional genomic context. Almost all of these sites are found in regulatory or non-coding regions within or close to genes (Figure 6B), in support of the earlier notion that DCC interactions with coding sequences are of lower affinity.

As the majority of DCC binding sites map to transcriptionally active parts of the X-chromosomal chromatin, we tested the transcription status of the HAS. Only 60% of these sites overlap with regions of elongating RNA polymerase II (data not shown). Taken together with the fact that there is also considerable binding to regions proximal to the transcription units (Figure 6B), we conclude that a substantial fraction of the HAS are not transcribed.

### High-Affinity Sites of the DCC Are Frequently Depleted of Nucleosomes

An important question regarding the strongest DCC binding sites is whether they contain common chromatin features that may help to explain X-chromosomal specificity. Previously, several of the few known HAS were shown to reside within regions of nucleosome depletion (DNase I hypersensitive sites) [15],[23],[24]. In order to explore whether this was a more general feature of HAS, we compared the location of the HAS with the published histone H3 profile [21]. Visual inspection of the data reveals that a large fraction of the sites colocalize with regions of low histone H3 content (see example in Figure 6A). In general, microarray probes located within HAS had significantly lower H3 signals than the ones outside (p-value<2.2e-16; two-sided t-test). Calculating the cumulative H3 distribution across all 131 HAS (Figure 6C) we found that the H3 profile clearly dropped towards the centers of the sites in a window of about 1 kb. The low resolution of the analysis based on the rather large chromatin fragments generated in our ChIP procedure (500 bp) does not allow for a robust determination of the number of nucleosomes or the length of DNA that might be affected. Clearly, however, nucleosome depletion is not alone sufficient to initiate DCC binding, as the number of X-chromosomal nucleosome depleted regions (1148) greatly exceeds the number of HA sites.

### Sequences Comprising Dinucleotide Repeats Characterize the High-Affinity Sites

**Figure 7:**
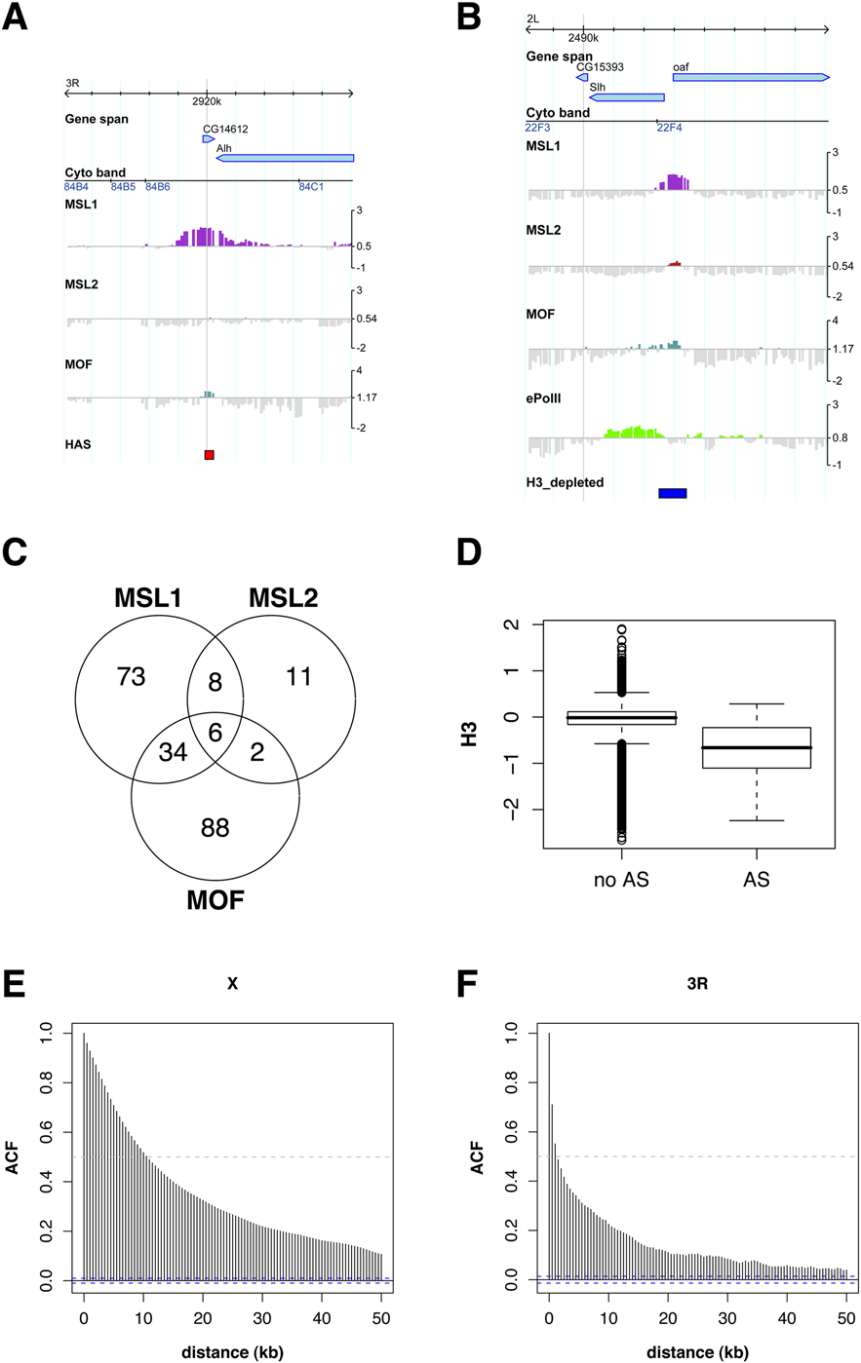
Autosomal binding sites of the DCC. A) Autosomal high affinity site. B) Autosomal site bound by MSL1, MSL2 and MOF. C) Venn diagram showing the colocalization of MSL1, MSL2 and MOF on autosomal binding sites. D) Boxplot comparing histone H3 signal on probes located on autosomal binding sites (AS) and probes located elsewhere on autosomes. E) Autocorrelation (ACF) of MSL1 binding to the X chromosome and (F) to chromosome 3R. Correlation was performed on smoothed profiles with 500 base spacing.

The low nucleosome occupancy of the HAS suggests that primary recruitment of the DCC to the X chromosome may involve recognition of exposed DNA sequences rather than histone modifications. We therefore performed extensive sequence analysis in order to identify motifs that are significantly enriched in the strongest binding sites. A motif that was identified most robustly in several MEME [25] analyses with varying parameters and training sets is shown in Figure 7d. An example MEME run on the strongest of our HAS is provided in the supplement (Dataset S1). Results varied depending on the size of the training set and the analysis parameters. However, dinucleotide repeats based on either GA or CA as well as runs of adenines were frequently identified. Such sequences have already been postulated to be involved in DCC recruitment [10],[13],[22],[24]. The GA-repeat based motif shown in Figure 6D is the only one that was present in a large fraction of HAS (68 of 131 by MAST analysis) and at the same time enriched on the X chromosome (1.5 fold by genome-wide search with the MEME-derived position-specific scoring matrix). Only 33% of the motifs identified on the X chromosome are, however, bound by the complex in SL2 cells, suggesting we may be missing the context within which the identified sequence motif might contribute to DCC recruitment. For example, the site may operate in conjunction with a variable cohort of secondary motifs, as suggested by previous fine-mapping of known HAS elements [24].

### Autosomal MSL Binding Sites Resemble X Chromosomal Targets, but Bind MSL Proteins Locally Restricted and With Non-DCC Stoichiometry

Interestingly, the single autosomal site that was picked up at the chosen threshold by our approach binds MSL1 robustly in the absence of significant amounts of MSL2 (Figure 7A). This is remarkable considering the widely held belief that MSL1 and MSL2 mutually depend on each other for chromosome interaction [19],[26]. To address the stoichiometry of DCC subunits at autosomal sites more systematically we identified all binding sites for MSL1, MSL2 and MOF statistically and found that these three proteins do not colocalize on most sites, with MSL2 showing the lowest occupancy (Figure 7C). Intriguingly, on autosomes MSL1 mainly binds to promoters of active genes (as seen e.g. in Figure 7B). These are depleted of nucleosomes, in analogy to X chromosomal HAS: probes within the autosomal binding sites show significantly reduced histone H3 content (p-value = 3.4e-14; two-sided t-test). Sequence analysis of the autosomal sites with strongest MSL1 binding revealed dinucleotide repeats (mainly GA and CA) similar to the ones that characterize the X chromosomal HAS (Datasets S1 and S2). One notable difference between MSL1 binding sites on the X and on autosomes is that whereas X chromosomal HAS usually display a rather broad MSL1 distribution, MSL1 binding at autosomal sites is spatially restricted and does not spread substantially onto the adjacent active chromatin, even at sites where all tested complex components colocalize (Figure 7B). This is confirmed globally by the autocorrelation of MSL1 binding on a smoothed profile with a spacing of 500 bases, which shows that binding domains on the X are much broader than those on autosomes [compare Figures 7E and F, autocorrelation (ACF) of >0.5 within 10 kb (X) or of 0.5 at a maximum of 2 kb (3R)], the latter most likely reflecting rather restricted or singular binding events blurred by the ChIP resolution of about 500 bases. Furthermore, MSL1 binding to autosomal sites not only remains upon reduction of MOF, MSL3 or MLE through RNAi, but is even increased under those conditions (Figure S3). This might reflect a re-distribution of MSL sub-complexes from the X to autosomes after elimination of the spreading component.

### Chromosomal Organization of Dosage Compensation

**Figure 8:**
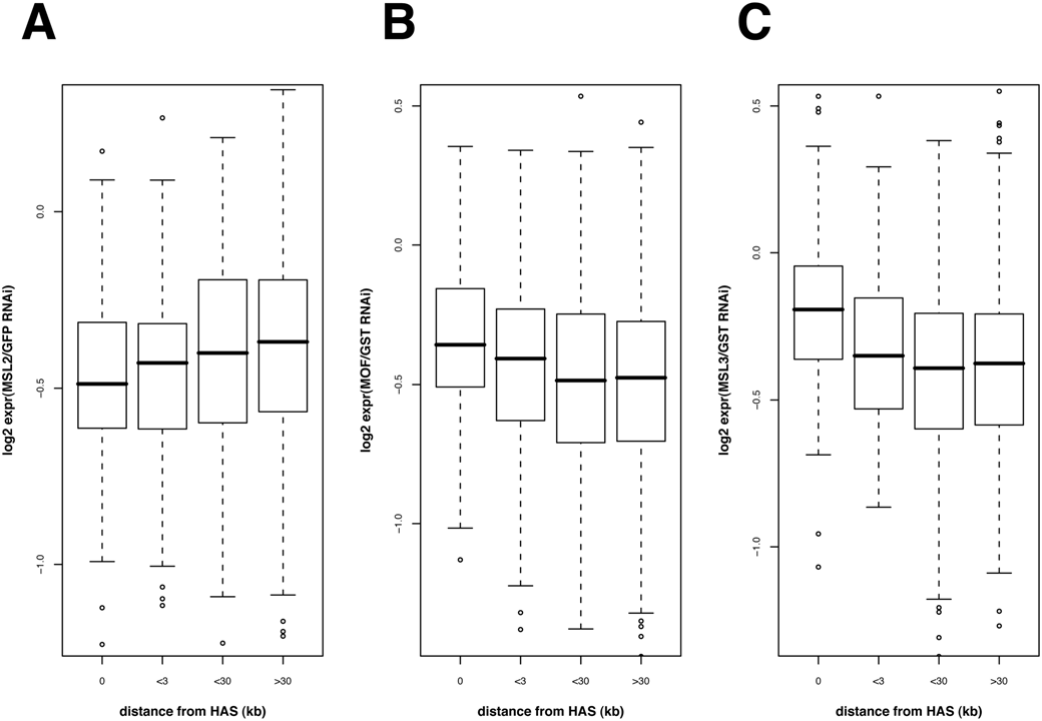
Compensation of X-linked genes is dependent on distance from high-affinity sites (HAS). Based on microarray expression profiling studies the relation of mean fold changes (log2) in gene expression after RNAi and the genes' distance from the closest HAS is depicted. A distance of 0 indicates that the HAS is directly within the gene. Effects of MSL2 (A), MOF (B) and MSL3 (C) knockdown are displayed as boxplots on gene groups of varying distance. For clarity, extreme outliers have been omitted from the panels. Genes located more than 3 kb away from the next HAS respond significantly less to MSL2 RNAi (p-value 0.00087; two-sided t-test). On the contrary, upon MOF and MSL3 RNAi these genes are stronger affected (p-value 3.115e-05 and 5.804e-08, respectively).

The 130 HAS defined by our analysis are distributed all along the X chromosome with a preferred distance between 60–300 kb. If the HAS were the primary organizers of larger dosage compensation domains, we would expect a relationship between the robustness of transcriptional compensation of genes and their distance from the nearest sites. Figure 8 shows that this is actually the case. Dosage compensation reflected by the drop of transcript upon ablation of MSL2 by RNAi decreases with growing distance (Figure 8A). On the other hand, the further away a given gene is from a HAS, the more dependent is its compensation on the spreading factors MOF and MSL3 (Figures 8B & C). This finding demonstrates that the HAS we have identified play a role as organizers of compensated domains.

## Discussion

Combining differential crosslinking and RNAi interference against the DCC subunits previously shown to be required for the ‘spreading’ of the complex from high affinity or ‘entry’ sites we identified 131 high-affinity sites (HAS) of the *Drosophila* dosage compensation complex in male SL2 cells. This set of sites contains all previously identified HAS (or chromosomal entry sites, CES) and a representative selection colocalizes with interbands on polytene chromosomes that had been described as harboring primary binding sites for the DCC in previous genetic analyses. The sites we now identified thus have similar properties to the ones identified by genetic means. Our study not only provides a much large number of such sites, but also resolves their positions and widths much more precisely than enabled by the polytene chromosome analyses. Most importantly, our study suggests that the HAS have a function in dosage compensation since we observe a positive correlation between the proximity of genes to a HAS and the extent of dosage compensation. Conversely, the further away genes reside from the nearest HAS the more they depend on the spreading factors such as MOF or MSL3 for enhancement of transcription. The 130 X-chromosomal HAS are distributed all along the chromosome with a predominant spacing between 60 and 300 kb. The realm within which loci profit from the presence of a high affinity ‘DCC attraction center’ may be of the same order of magnitude. However, we generated the inventory of HAS by applying fairly stringent thresholding criteria. Less stringent selection criteria will undoubtedly reveal a large number of sites with degenerate features and lower affinities that may serve as ‘relay stations’ for DCC spreading and may contribute cumulatively to concentration of the DCC on the X chromosome[27]. Finally, the linear display of DCC–chromosome interactions in a browser obviously does not reflect the three-dimensional path and packaging of the chromosomal fiber, which might facilitate transfer of a chromatin-bound complex between distant loci.

Under normal circumstances the DCC binds with high preference to transcribed and, indeed, coding sequences [9],[10]. Our observation that a transcribed region upstream of the *Nej* gene harbors a strong site in our set of binding sites but is not occupied in polytene chromosomes may, therefore, be due to differences in the transcription status between salivary glands and SL2 cells. Selection for sites of higher affinity leads to preferential loss of DCC from coding sequences, and under low-crosslinking conditions the majority of DCC binds at non-coding sequences in UTRs, introns, and also outside of the transcribed sequences in presumed regulatory and intergenic regions. Apparently, coding sequences have a lower affinity than non-coding sequences. At least part of the attraction of the DCC to transcribed sequences is due to the histone H3K36me3 mark, which is co-transcriptionally placed by Set2 and may provide a docking site for MSL3 [21]. However, this modification marks all transcribed sequences on autosomes as well and cannot be responsible for primary targeting. If, as suggested by this and previous work [10],[13],[24],[28], DNA sequence motifs contribute to DCC targeting, the observed preference for HAS outside of coding regions makes sense: assuming that binding affinity increases as sites conform with an idealized ‘consensus’ sequence, evolution of HAS with better defined sequences will be limited at coding regions where the main selective pressure is on preserving protein coding. If coding regions contain sequence elements that bind DCC they may, therefore, be of lower affinity and hence be preferentially lost as the stringency of the selection increases.

Sequence analysis of the HAS did not lead to the identification of a single motif that could explain the HAS interaction pattern. Rather, we found low complexity sequences, in particular GA and CA dinucleotide repeats, generally enriched in HAS, but in no instance present in more than 50% of the sites. The results of the sequence analysis fluctuate considerably depending on the selected training set, the analysis parameters and algorithms used. The only motif that was found consistently within the set of HAS that is also enriched on the X chromosome is an almost perfect 11mer of GA. We previously identified similar repeats employing very different strategies [10],[13]. Blocks of GA are also important for targeting the DCC to a nucleosome-free region within the roX2 gene [22].

Recently, Kuroda and colleagues published a similar study including high resolution mapping of HAS of the Drosophila DCC [29]. Even though they used *Drosophila* embryos and different experimental approaches (e.g. genetic knockouts instead of RNAi and Solexa sequencing in addition to tiling array analysis) the results of the two studies match surprisingly well. In fact, 90 of our 130 X-chromosomal HAS perfectly overlap with the chromosomal entry sites (CES) from the Kuroda lab (the differences in sites may well be explained by the different transcriptional status of the cells/embryos employed in the two studies). The GA-based sequence motif that we found enriched in the HAS perfectly covers the consensus MSL response element of the Kuroda lab and they also observe a comparable histone depletion among their HAS. Using a reporter gene assay a role for the GA-rich sequence element in transcription activation was documented [29]. This not only confirms the suitability of our experimental approach but also reveals that a large fraction of HAS overlap in different specimens.

How GA repeat motifs contribute to DCC loading is not known, but several scenarios may be considered. So far, a direct interaction of DCC subunits with specific DNA elements cannot be excluded. Further, DCC targeting may rely on interaction with an accessory protein with appropriate sequence preference, such as Pipsqueak or the GAGA factor (GAF) encoded by the *Trithorax-like* (*Trl*) gene. These two GAG-binding proteins colocalize at numerous sites on polytene chromosomes [30]. Hypomorph *trl* mutants show a male-specific lethality if the levels of MSL1 and MSL2 are reduced [31]. However, GAF only colocalizes with MSL2 at one out of 33 HAS and mutant larvae with strong *Trl* alleles show no obvious alteration of the DCC binding pattern on polytene X chromosomes. However, they display an increased number of autosomal binding sites, which may indicate a certain perturbation of targeting [31]. GA-rich elements may synergize with other DNA sequences (and hence other interacting factors) to form HAS, as previously suggested [13],[24]. Local clustering of two unrelated DNA sequence motifs, neither of which is particularly enriched on the X chromosome, appears to be crucial for targeting the DCC in *C. elegans* [32].

The affinity of a given DNA sequence for an interacting factor is strongly lowered by its nucleosomal organization [27]. Chromatin serves as a general thresholding system to present only those binding sites that reside in an appropriate non-nucleosomal context or benefit from nucleosome remodeling [33]. Interestingly, we find that the HAS, independent of whether they are located in regulatory regions, introns or outside of transcribed sequences, tend to be depleted of nucleosomes. Nucleosome depletion alone is not a stringent determinant of DCC association since many sites of low nucleosome density do not contain HAS or are not bound by the complex. Conversely, not all HAS are entirely nucleosome-free. Nevertheless, an improved definition of HAS may require considering the degree of nucleosome occupancy of sites in addition to the actual sequence itself. Nucleosome disruption may be brought about by ATP-dependent nucleosome remodeling or by competition of DCC binding with nucleosome assembly at the replication fork [34]. In the latter scenario the absence of nucleosomes would be a consequence of DCC binding rather than a requirement for interaction. Nucleosomes are also disrupted by the progression of the elongating RNA polymerase, a fact that may explain the recent observation that DCC binding to a sequence element within the MOF gene benefited from transcription [28].

Dinucleotide repeats and nucleosome depletion are also characteristic of autosomal MSL binding sites, however, these sites differ from HAS by two interesting features. First, we observed an altered stoichiometry of MSL proteins at autosomal sites, which often appear to lack MSL2. At these sites the colocalization of MSL1, MSL2 and MOF is the exception rather than the rule, suggesting that the known interdependence of MSL1 and MSL2 for chromosome association [19] is not absolute, but context-dependent. Second, binding of MSL proteins to autosomal sites appears unusually confined and does not spread onto the adjacent active chromatin as is commonly observed for X-chromosomal HAS. Lack of spreading is also found in the presence of MSL2. Because the distribution of MSL proteins from initial targeting sites is strongly facilitated by transcription of roX RNA from the same chromosome [11],[21],[35], we speculate that autosomal sites may be bound by MSL proteins in the absence of roX RNAs.

Our data are consistent with a multi-step model of X chromosomal targeting by the DCC, which involves assembly of the complex with nascent roX RNA within the X chromosomal territory, followed by its diffusion to and concentration by the set of HAS, which we have identified in this study. Distribution to all target genes may then be brought about by large numbers of low affinity sites and the transcription-associated H3K36 methyl mark.

## Materials and Methods

### *Drosophila* Cell Culture and RNA Interference

Cultivation of the male *Drosophila* cell line SL2 and RNA interference of target genes were carried out as described previously [36]. In brief, 1×10^6^ SL2 cells were incubated with 10 µg dsRNA for 1 hour in serum-free medium. After addition of serum-containing medium, cells were incubated for 7 days at 26°C before chromatin preparation. Preparation of whole cells extracts and western blot confirmation of target gene knockdown has been described previously [36]. Depletion efficiency was quantified using a Li-Cor Odyssey system using α-tubulin as a reference. Sequences of primers used for dsRNA production are listed in Table S2.

### Chromatin IP

SL2 cells were crosslinked in growth medium using 1% formaldehyde for 60 minutes in icewater. Alternatively we used 0.1% formaldehyde for 10 minutes at RT (low formaldehyde crosslinking). Fixation was quenched by addition of glycine to a final concentration of 125 mM. After washing, cells were resuspended in RIPA buffer and sonicated using a Bioruptor (Diagenode, Belgium) 8 times 30 seconds using the ‘high’ setting. Fragment size of the obtained chromatin was checked to be between 300 bp and 700 bp. Chromatin was precleared using a protein A/protein G-sepharose mixture for 1 hr at 4°C. 200 µl chromatin was incubated with appropriate amounts of antibodies in a total volume of 500 µl RIPA buffer at 4°C overnight. After washing and crosslink reversal, immunprecipitated nucleic acids were purified on GFX columns (GE Healthcare). Input chromatin serving as reference sample was treated accordingly. Overall, we performed immunoprecipitations for MSL1 (4 biological replicates) and MSL2 (2 replicates) on chromatin from untreated SL2 cells. In addition, we precipitated MSL1-containg chromatin after GST, MSL3, or MLE RNAi (2 replicates each). After low formaldehyde crosslinking, we performed ChIP for MSL2 from untreated cultures (2 replicates) and MSL1 IP after GST or MOF RNAi (3 replicates each). The rabbit polyclonal MSL1 and MSL2 antibodies used in this study were described elsewhere [10],[24].

### Tiling Array Analysis

Input and IP DNA were amplified using the WGA kit (Sigma) according to an online protocol (http://www.epigenome-noe.net/researchtools/protocol.php?protid30). Labeling and hybridization to NimbleGen arrays was carried out at ImaGenes (Berlin, Germany). We used a custom array layout (approx. 1 probe/100 bases) comprising the euchromatic part of the entire X chromosome, 5 MB of 2L, 2R and 3L, respectively, as well as 10 MB of 3R. Data analysis was performed using R/Bioconductor (www.R-project.org; www.bioconductor.org). Raw signals of corresponding experimental replicates were normalized using the ‘vsn’ package [37]. Enrichment statistics (IP versus input signals) were computed using the ‘sam’ algorithm within Bioconductor [38]. Fdr values of the sam statistic were determined using ‘locfdr’ [39]. Region summarization was performed using the HMM algorithm of TileMap [40]. Probes were considered to be bound significantly if the posterior probability of the HMM was greater than 0.5. Statistical tests and presentations were performed using R defaults if not indicated otherwise. Details about high-level computations are available upon request. Visualization was carried out by loading the mean enrichment ratios as GFF files into GBrowse (www.gmod.org). All data correspond to *Drosophila* genome version dm2 and annotation version gadfly 4.3. Raw data was deposited at the NCBI gene expression omnibus, GEO (data series GSE12292). Wild type profiles and locations of high-affinity sites are available for browsing at http://genome1.bio.med.uni-muenchen.de.

### Additional Data Sources

The histone H3 profile and regions of histone depletion in SL2 cells were calculated from the GEO data series GSE8557 [21]. Gene expression changes upon RNAi of MSL2 in SL2 cells were derived from [41]. MOF and MSL3 knockout data were downloaded from ArrayExpress, accession E-MEXP-1505 [42].

### Immuno-FISH on *Drosophila* Polytene Chromosomes

FISH probes spanning the selected high-affinity sites were PCR amplified from genomic DNA. Primer sequences for the individual probes are listed in the supplement (Table S2). Immuno-FISH was performed exactly as described online (http://www.epigenome-noe.net/researchtools/protocol.php?protid4).

## Supporting Information

Figure S1 MSL3 and MLE RNAi reduce MSL1 binding to coding sequences. A) Genome browser snapshot with gene spans and gene models. MSL1 binding profiles after GST, MSL3 and MLE RNAi are provided. Depicted is the log2 of the mean enrichment ratio (IP/Input) of 2 replicate experiments. B) Barplot showing the relative distribution of probes significantly bound by MSL1 after GST, MLE and MSL3 RNAi with respect to functional genomic context. Proximal probes are defined as those located within 500 bases up- or downstream of genes. C) Boxplot of changes in MSL1 enrichment after MLE and MSL3 RNAi on MSL1 target probes. Colour grouping of boxes corresponds to functional context. The left box of the duplicates corresponds to MLE RNAi , the right one to MSL3 RNAi.
(1.78 MB TIF)

Figure S2 MSL1 binding is resistant to RNAi at high-affinity sites: Boxplots of probe-level MSL1 enrichment changes in MSL1 binding regions after RNAi divided into HAS and non-HAS probes for different RNAi experiments. P-values of two-sided t-tests are provided.
(0.40 MB TIF)

Figure S3 Autosomal MSL1 sites are resistant to MOF RNAi. A) Absolute changes in the number of autosomal probes that are significantly enriched in MSL1 and (B) the corresponding relative changes. C) Differences in MSL1 signal on MSL1 target probes after MOF RNAi grouped by functional context.
(0.30 MB TIF)

Table S1 List of all high-affinity sites identified by our approach.
(0.03 MB XLS)

Table S2 Sequences of primers for generation of dsRNA and primers for FISH probe productions.
(0.02 MB XLS)

Dataset S1 Exemplary MEME analysis output of the top 30 high-affinity sites.
(0.20 MB DOC)

Dataset S2 Exemplary MEME analysis output of the top 20 autosomal MSL1 binding sites.
(0.20 MB DOC)
