# Supplementary figures and images for: The Chromosomal High-Affinity Binding Sites for the Drosophila Dosage Compensation Complex
Source: PLoS Genet. 2008 Dec 12;4(12):e1000302. doi: 10.1371/journal.pgen.1000302 (PMC2586088; doi:10.1371/journal.pgen.1000302)

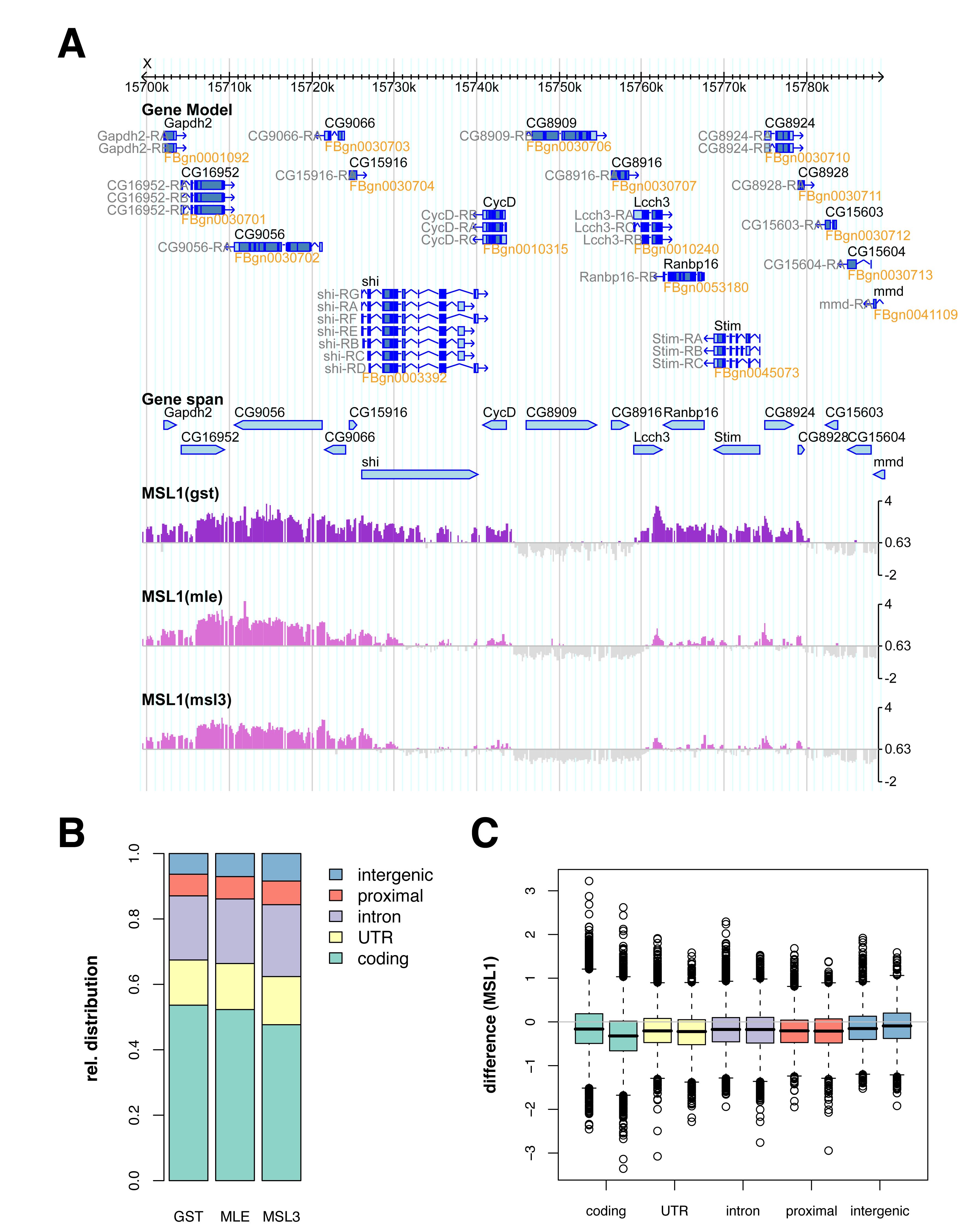

Supplement: Figure S1 — MSL3 and MLE RNAi reduce MSL1 binding to coding sequences. A) Genome browser snapshot with gene spans and gene models. MSL1 binding profiles after GST, MSL3 and MLE RNAi are provided. Depicted is the log2 of the mean enrichment ratio (IP/Input) of 2 replicate experiments. B) Barplot showing the relative distribution of probes significantly bound by MSL1 after GST, MLE and MSL3 RNAi with respect to functional genomic context. Proximal probes are defined as those located within 500 bases up- or downstream of genes. C) Boxplot of changes in MSL1 enrichment after MLE and MSL3 RNAi on MSL1 target probes. Colour grouping of boxes corresponds to functional context. The left box of the duplicates corresponds to MLE RNAi , the right one to MSL3 RNAi. (1.78 MB TIF) [file pgen.1000302.s001.tif]

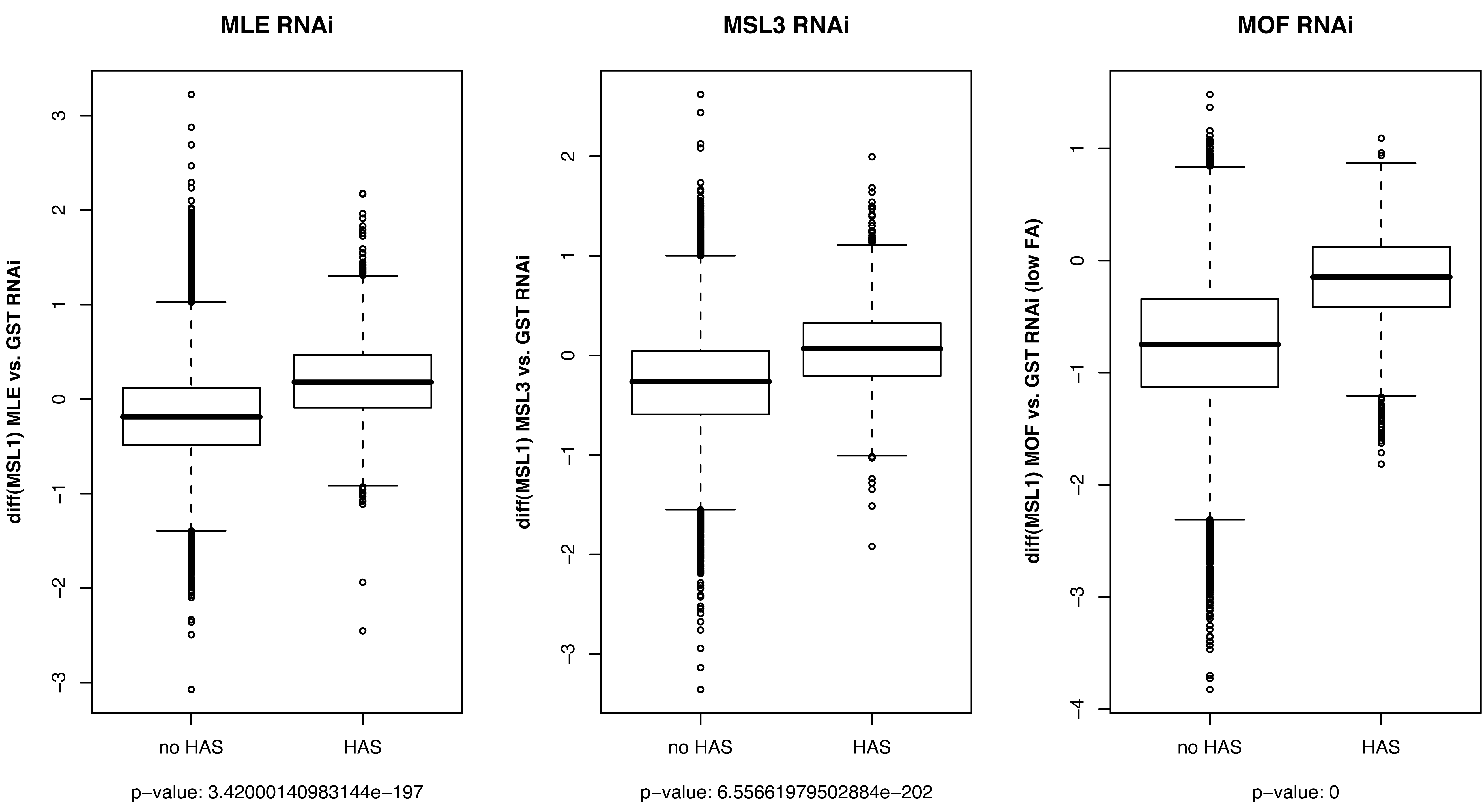

Supplement: Figure S2 — MSL1 binding is resistant to RNAi at high-affinity sites: Boxplots of probe-level MSL1 enrichment changes in MSL1 binding regions after RNAi divided into HAS and non-HAS probes for different RNAi experiments. P-values of two-sided t-tests are provided. (0.40 MB TIF) [file pgen.1000302.s002.tif]

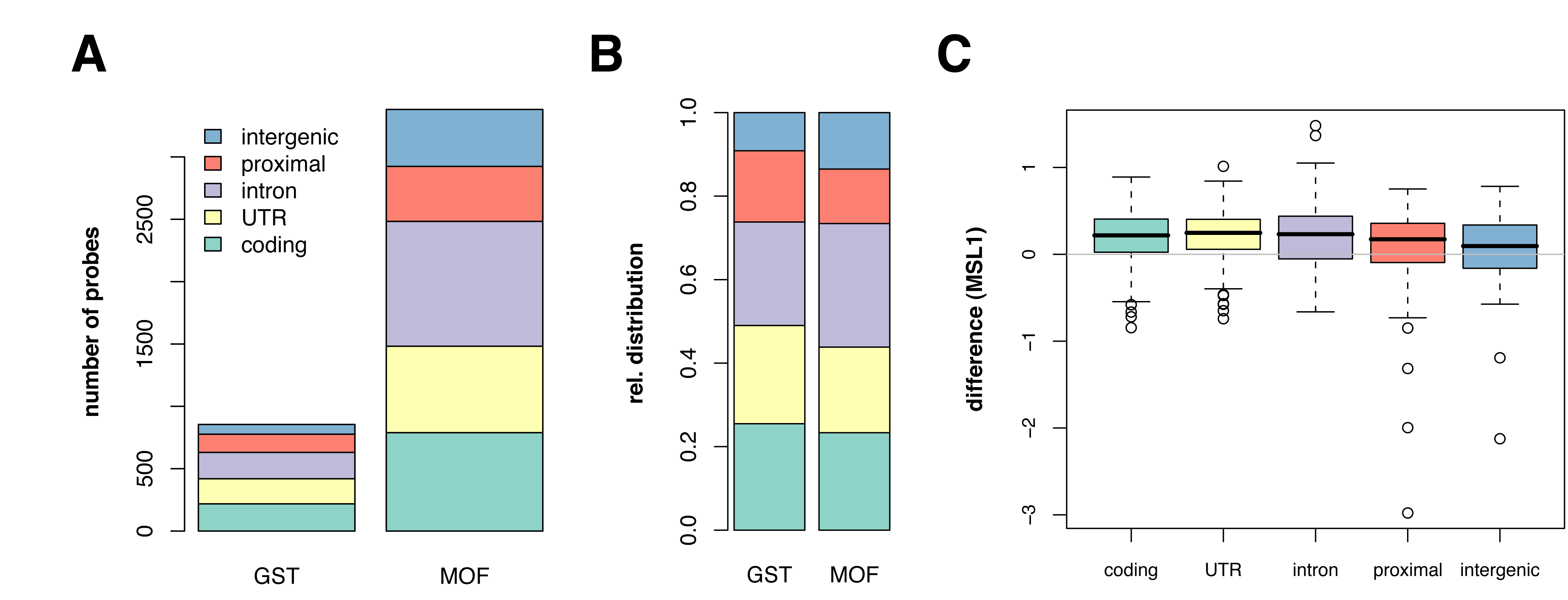

Supplement: Figure S3 — Autosomal MSL1 sites are resistant to MOF RNAi. A) Absolute changes in the number of autosomal probes that are significantly enriched in MSL1 and (B) the corresponding relative changes. C) Differences in MSL1 signal on MSL1 target probes after MOF RNAi grouped by functional context. (0.30 MB TIF) [file pgen.1000302.s003.tif]
